# Safety, Efficacy, and Intermediate‑Term Outcomes of CT‑Guided Cryoablation of T1 Renal Cell Carcinoma: A Retrospective Single‑Center Study

**DOI:** 10.5334/jbsr.3919

**Published:** 2026-03-11

**Authors:** Laura Frenoy, Pieter‑Jan Buyck, Lawrence Bonne, Ben Van Cleynenbreugel, Maarten Albersen, Geert Maleux

**Affiliations:** 1 Department of Radiology, University Hospitals KU Leuven, Leuven, Belgium; 2 Department of Urology, University Hospitals KU Leuven, Leuven, Belgium

**Keywords:** renal cell carcinoma, computed tomography, percutaneous cryoablation, follow‑up, survival

## Abstract

*Objectives:* To assess the safety, efficacy, and mid‑term clinical and radiological outcomes of computerized tomography (CT)‑guided cryoablation of malignant T1 renal lesions.

*Methods:* Consecutive patients who underwent percutaneous cryoablation (PCA) under CT guidance between January 2018 and January 2024 for one or multiple renal masses were included. Technical success and primary and secondary treatment efficacy were calculated. Complications were categorized according to the Clavien–Dindo classification system. Overall survival (OS) and local progression‑free survival (LPFS) were assessed based on Kaplan–Meier analysis.

*Results:* A total of 72 renal lesions, with a mean size of 23.4 mm (SD: 9.8, range 7–45), were treated in 59 patients (41 males and 18 females, mean age: 69 years [range 45–88]). A total of 25 patients (42.4%) had a history of radical and/or partial nephrectomy. Primary treatment efficacy was 91.0% and increased to 95.6% with the re‑ablation of two lesions. The overall complication rate was 10.8%, with a major complication (Clavien–Dindo grade ≥ III) rate of 3.1%. The estimated LPFS at 15 months was 92.9%. Local progression occurred in four lesions, of which two had already been ablated. The estimated OS at 1 year was 95.9% (standard error [SE] of 2.8%) and 91.6% (SE: 5.0%) at 3 years.

*Conclusion:* Percutaneous CT‑guided cryoablation is a safe and effective treatment for small renal cell carcinoma.

## Introduction

Renal cell carcinoma (RCC) represents approximately 90% of all renal tumors, with a worldwide increase of 2% in the last 20 years [1], in particular, an increase in the incidental findings of small renal masses [2, 3]. Surgery is still considered the standard curative treatment, with partial nephrectomy (PN) being the treatment of choice for T1 RCC (≤7 cm) [1, 4]. Unfortunately, not all patients are suitable candidates for surgery related to comorbidities such as chronic obstructive lung disease, heart failure, advanced age, end‑stage renal disease, and/or the presence of a solitary kidney. In addition, redo PN‑surgery may be technically challenging due to the presence of in situ fibrosis and scarring of normal tissue planes [5]. Moreover, complications such as pleural injuries, pancreatic leakage, renovascular injuries, and urine leaks are more prevalent in repeated PN procedures [5, 6]. Last, higher complication rates also apply to repeat robotic PN [5]. Given these conditions, a less intrusive approach using percutaneous image‑guided thermal ablation gained popularity, with radiofrequency ablation and cryoablation emerging as the primary modalities. Even though a meta‑analysis showed similar efficacy and complication rates, cryoablation is often preferred due to the visible formation of the ice ball on CT imaging and subsequently, the visualization of the ablation margin [7].

The initial studies demonstrated that percutaneous cryoablation (PCA) showed a low rate of major complications, ranging from 3.4% to 12%, and no significant difference in renal function before and after intervention [8–13]. Thereafter, studies with oncological outcomes started to emerge. Two meta‑analyses have reported similar oncological outcomes between thermal ablation and PN surgical treatment [14, 15]. Other recent studies confirm these findings [16, 17]; however, according to the 2022 European Association of Urology (EAU) Guidelines on RCC, ‘the quality of the data available does not allow any deﬁnitive conclusions regarding morbidity and oncological outcomes’ [1]. This led the EAU to indicate thermal ablation in the following cases: small renal masses in elderly, comorbid patients unfit for surgery, genetic predisposition to develop multiple tumors with recurrences after previous surgery, bilateral tumors, solitary kidney, and finally, cases with a high risk of complete loss of renal function following PN [1].

The present study aimed to retrospectively assess the safety and efficacy of CT‑guided PCA for T1 RCC in a consecutive group of selected patients, treated in a Belgian, tertiary, academic care center for urological oncology.

## Materials and Methods

### Study design and population

This retrospective, single‑center study was approved by the Ethics Research Committee of the University Hospitals Leuven (MP023461). The institutional Interventional Radiology database was searched for consecutive patients who underwent CT‑guided PCA in the authors’ institution between January 2018 and January 2024 for the management of one or more small renal masses. Patients with a biopsy‑proven renal tumor or a renal mass with suspicious characteristics and increased risk for biopsy‑related hemorrhage were discussed in a multidisciplinary oncologic consult (MOC), including urologists and radiologists. The PCA procedure was planned if the MOC considered percutaneous treatment as the most suitable option and if the interventional radiologist considered the PCA procedure as technically safe and feasible based on preinterventional contrast‑enhanced computed tomography and/or magnetic resonance imaging (MRI), and if the patient agreed with the proposed treatment algorithm.

### PCA procedure

All PCA procedures were performed by experienced interventional radiologists (PB, LB, GM), under general anesthesia with the patient in the prone position on a CT table. First, a non‑contrast CT scan was performed. If no clear visualization of the tumor could be obtained, an additional contrast‑enhanced CT scan was performed. Under fluoro‑CT‑guidance, one or more cryoprobes (Iceforce, Boston Scientific, Marlborough, MA, USA) were placed to completely cover the tumor. Freezing of the tumor was achieved by using an Argon gas cryoablation. When an organ was considered too close to the margin of the tumor, carbo‑dissection (CO_2_‑Angioset, Optimed, Ettlingen, Germany) was used to keep the neighboring organ at a safe distance from the ice ball. This was achieved by placing a 22‑gauge needle (Chiba needle, Cook Medical, Bloomington, IN, USA) to insufflate CO_2_ in the retroperitoneal space between the organ at risk and the renal tumor. Once the cryoprobes were correctly placed, as confirmed by a control CT‑scan, the cryoablation protocol started. It consisted of a first 10‑minute freeze cycle, followed by a passive 8‑minute thaw, and a second 10‑minute freeze cycle. The second freeze cycle was followed by an active thaw in order to extract the cryoprobe(s).

After the first and second freeze cycles, a non‑contrast renal CT was performed for the visualization of the ice ball, completely covering the target tumoral lesion. Last, a final, non‑contrast CT was performed after removing the cryoprobe(s) for visualization of any procedure‑related complications, including retroperitoneal hemorrhage or pneumothorax. Technical success was defined as treatment according to the protocol and the ice ball completely covering the tumor with a minimal margin of 5 mm around the expected tumor margin [18].

In case of a post‑procedural bleeding complication, the patient was transferred to the institutional interventional radiology suite for immediate embolization. Under general anesthesia, the right common femoral artery was punctured, and a 4 French Cobra or Simmons 1 catheter (GlideCath, Terumo Europe, Leuven, Belgium) was used to cannulate the injured renal artery. Superselective catheterization of the bleeding end‑branches was performed through a microcatheter (Progreat 2.4, Terumo Europe, Leuven, Belgium) and microcoils (MicroTornado, Cook Medical, Bloomington, IN USA or Target microcoils, Boston Scientific, Natick, NY, USA) were used to embolize the injured renal arterial branches.

### Data collection

Demographic data, including patient’s age, sex, number of kidneys, comorbidities, history of radical or PN, and use of anticoagulants, were extracted from the patients’ electronic medical records. Comorbidities were classified using the Charlson Comorbidity Index. Additionally, the following comorbidities were documented: active smoking or history of smoking, diabetes mellitus type 1 or 2, arterial hypertension, chronic obstructive pulmonary disease, and chronic kidney disease. Chronic kidney disease stages as defined by Kidney Disease Improving Global Outcomes (KDIGO) were used [19].

Laboratory data included hemoglobin value, creatinine level, estimated glomerular filtration rate (eGFR), white blood cell (WBC) count, C‑reactive protein, activated partial thromboplastin time (aPTT), and international normalized ratio (INR) value.

Last, RENAL and PADUA nephrometry scores were calculated, based on the preinterventional CT and/or MRI scans, in patients without polycystic kidney disease.

### Safety

Patients were admitted to the hospital overnight, followed by a blood test and an ultrasound examination the next day as per standard protocol. Ultrasound was conducted to rule out pseudoaneurysms or perirenal hemorrhage and arteriovenous fistulas. Complications were classified following the Clavien–Dindo classification [20, 21]. A complication that required intervention or changed the treatment plan was considered clinically significant.

### Follow‑up

Primary treatment efficacy was defined as any absence of viable (= contrast enhancing) tissue within or adjacent to the ablation zone on the three‑month follow‑up CT scan. Secondary treatment efficacy was calculated for the re‑ablations.

Local tumor recurrence was defined as the appearance of a new enhancing nodule within or adjacent to the ablation zone on the follow‑up CT/MRI following previous negative imaging. Overall survival (OS) was defined as the period from the first PCA procedure to the date of death from any cause, with censoring of the patients still alive on January 31, 2024. Local progression‑free survival (LPFS) was defined as the period between the first PCA procedure and the date of the first local progression, with censoring of the patients without recurrence on the last CT or MRI data.

### Statistical analysis

Continuous data was described using mean ± standard deviation (SD) for data with normal distribution and mean and range for data with skewed distribution. The Shapiro–Wilk test was used to evaluate whether the continuous variables follow a normal distribution. Laboratory results were compared before and after PCA. This means that for the analysis of hemoglobin and eGFR, a paired *t*‑test was used. The Wilcoxon signed‑rank test was used for creatinine level and WBC count. LPFS was calculated per lesion and OS was calculated per patient. Kaplan–Meier analysis was used for OS and LPFS. A *p*‑value under 0.05 was considered statistically significant. All statistical analyses were performed using SPSS software, version 29.0.

## Results

### Patients and tumor characteristics

Of the 59 patients, 41 were male (69.5%) and 18 female (30.5%). The mean age at ablation was 69 years (SD: 10, range 45–88). The Charlson Comorbidity Index had a mean value of 6 (SD: 2, range 2–13). A total of 5 patients had a history of radical nephrectomy, 17 had a history of PN, and 3 had a history of both. Further, a tumoral lesion was found in a congenital, solitary kidney (*n* = 1), and in a horseshoe‑shaped kidney (*n* = 1). Chronic kidney disease was present in *n* = 32 patients, of which respectively *n* = 1, *n* = 3, *n* = 21, and *n* = 8 patients were considered in stadium I, II, III, and IV. The mean RENAL nephrometry score is 6.58 (SD: 1.857) and the mean PADUA score is 7.65 (SD: 1.687), based on 66 scored tumors.

Patients’ characteristics are summarized in Table 1.

**Table 1:** Patient characteristics.

PATIENT CHARACTERISTICS	*N*	%
Age		
Mean ± standard deviation	69 ± 10	
Range	45–88	
Sex		
Male	51	69.5
Female	18	30.5
Comorbidities		
Smoking or history of smoking	33	55.9
Arterial hypertension	43	72.9
Diabetes type I or II	15	25.4
Chronic obstructive pulmonary disease	7	11.9
Chronic kidney disease (CKD)	32	54.2
CKD stage		
1	1	1.7
2	3	5.1
3	21	35.6
4	8	13.6
5	0	0
Anticoagulant	30	50.8
Solitary kidney	10	16.9
Partial nephrectomy	20	33.9
Radical nephrectomy	8	13.6
Horseshoe kidney	1	1.7
Solitary kidney at birth	1	1.7
Polycystic kidneys	1	1.7
Von Hippel–Lindau	1	1.7

In total, 72 tumors were treated with 7 patients having 2 different tumors treated during single cryoablation session. The mean tumor size was 23.4 ± 9.8 mm. In *n* = 4 patients an intrarenal nodule of less than 1 cm diameter was treated; the indication to treat these lesions by PCA included: growth of the tumoral nodule with >5 mm over 6 months of follow‑up (*n* = 2) and appearance of a new, solid renal nodule in patients with a history of contralateral radical nephrectomy (*n* = 2). A total of 35 tumors were biopsy‑proven RCC of which 23 (65.7%) were clear cell RCC, as summarized in Table 2.

**Table 2:** Tumor characteristics.

TUMOR CHARACTERISTICS	*N*	%
Size (mm)		
Mean ± SD	23.7 ± 9.9	
Range	7–45	
Laterality		
R	37	51.4
L	35	48.6
Histology	72	
Clear cell RCC	23	31.9
Papillary RCC	8	11.1
Chromophile RCC	1	1.4
Chromophobe RCC	2	2.8
RCC with fibromatous stroma/RCC with leiomyomatous stroma	1	1.4
No biopsy of insufficient sample	37	51.4

R: right.

L: left.

RCC: renal cell carcinoma.

SD: standard deviation.

### Laboratory values

The median blood creatinine level before and after PCA was 1.15 mg/dL (range of 3.31) and 1.17 mg/dL (range of 3.56) respectively (*p* > 0.470); the eGFR before PCA (mean 59.14 ± 24.44 mL/min/1.72 m^2^) and the day after PCA (mean 57.34 ± 26.73 mL/min/1.72 m^2^) did not differ significantly either (*p* = 0.133).

Further, the hemoglobin value was a mean 13.85 ± 1.89 g/dL and a mean 12.70 ± 1.86 g/dL before and after PCA respectively (*p* < 0.001). Furthermore, the WBC count before and after PCA was a mean of 7.35/μL (range 22.56) and 10.19/μL (range 32.09) respectively (*p* < 0.001).

### Procedural data

**Figure 1:**
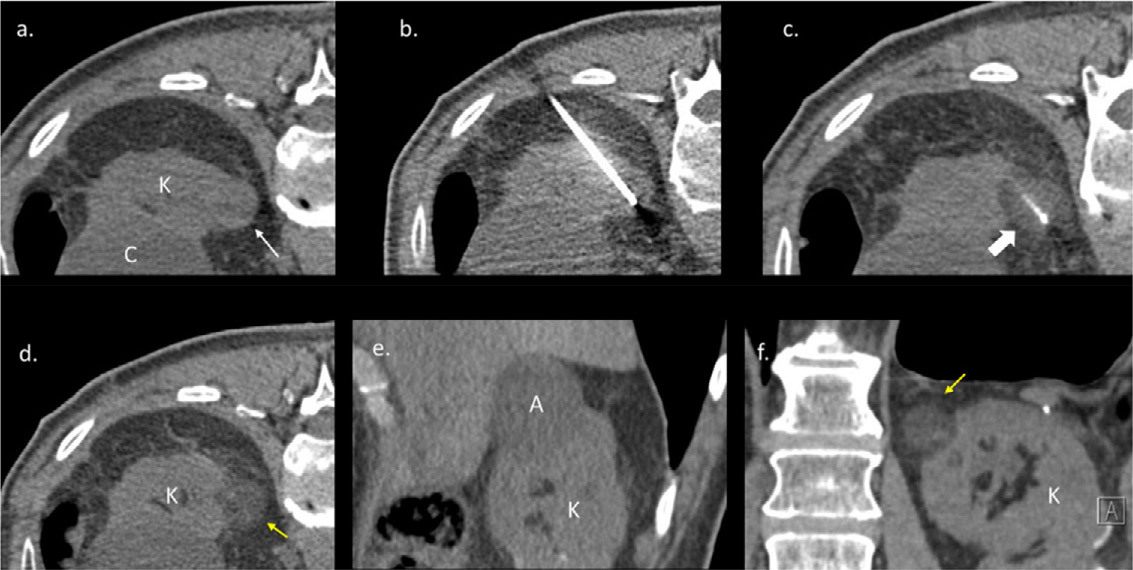
**a.** Non‑contrast axial CT scan before the cryoablation shows the exophytic RCC (thin white arrow) in the left kidney (K) and a big renal cyst (C). **b.** The cryoablation needle is inserted into the RCC through a posterolateral access route. **c.** Cryoablation needle in the RCC with a surrounding ice ball (thick white arrow). **d.** Non‑contrast axial CT scan after the procedure shows the ablated RCC (yellow arrow). **e.** Sagittal reconstruction CT scan shows the ablated RCC (A). **f.** Coronal reconstruction CT scan shows the ablated tumor (yellow arrow).

A mean of 1.9 cryoprobes were used per ablated tumoral lesion (Figure 1); carbo‑dissection was necessary in four PCA procedures. In *n* = 1 patient, the complete freezing time was not achieved related to depletion of Argon gas at the near end of the PCA‑procedure. However, the three months CT follow‑up could not demonstrate residual, viable tumoral tissue.

Procedure‑related complications were identified in *n* = 7 patients (10.8%) and major complications (Clavien–Dindo grade ≥ III) in *n* = 2 patients (3.1%). Small, clinically nonsignificant perirenal hematoma was found in *n* = 5 patients (7.7%) and a small, clinically nonsignificant perirenal fluid collection was seen in *n* = 8 patients (12.3%). Three patients (5%) experienced a decrease in hemoglobin (<1 mL/dL), but none of them needed a blood transfusion. No complication of Clavien–Dindo grade IV or more occurred.

**Figure 2:**
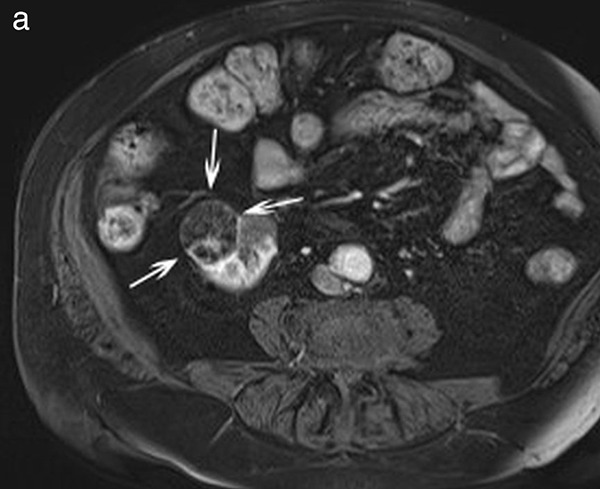

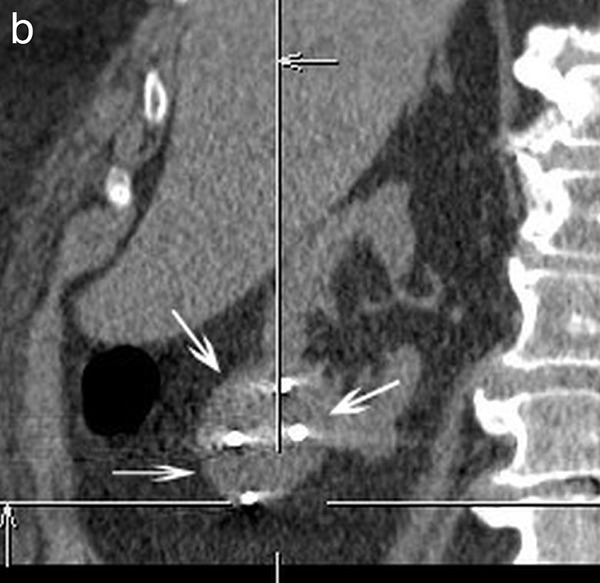

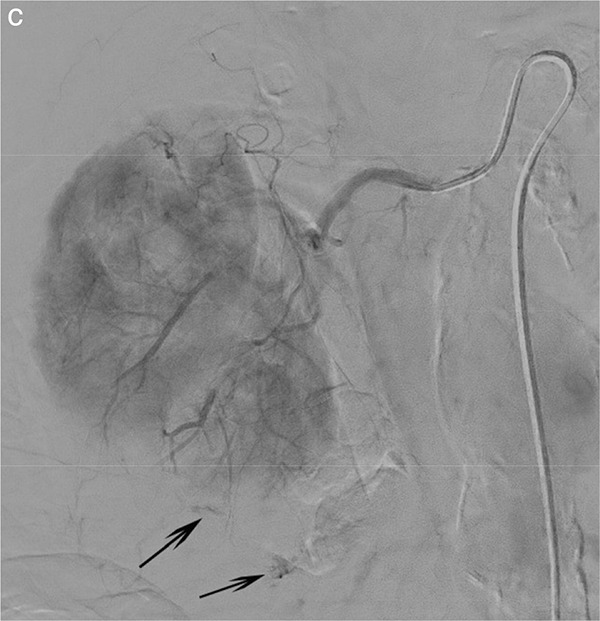

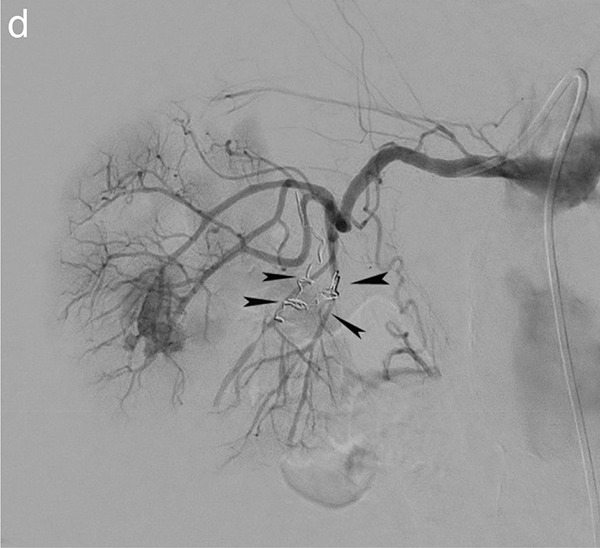
**a.** Contrast‑enhanced T1‑weighted MR‑image shows a hypovascular mass lesion (arrows) in the right anterior lower pole. **b.** Unenhanced, reconstructed parasagittal CT‑image during percutaneous cryoablation procedure shows exophytic mass lesion (arrows) and four needles within the mass lesion. **c.** Procedural retroperitoneal hemorrhage occurred and subsequent emergent selective angiography revealed two bleeding points (arrows) at the lower pole of the right kidney. **d.** Selective angiography after coil‑embolization (arrowheads) shows complete occlusion of the bleeding renal end branches.

The Clavien–Dindo grade III complications included formation of a large perirenal hematoma during the PCA‑procedure in one patient; this bleeding complication was subsequently and immediately managed with a coil‑embolization of multiple, extravasating terminal branches of the renal artery (Figure 2). The next day, the hemoglobin level decreased to 8.0 g/dL without further deterioration and no need for blood transfusion. Blood creatinine level increased from 2.12 mg/dL to a maximum 3.30 mg/dL 2 days post‑PCA and eGFR decreased from 22 mL/min/1.72 m^2^ to 13 mL/min/1.72 m^2^. Three months post‑PCA the patient presented at the emergency department with progressive symptoms of general malaise and weakness since the complicated PCA‑procedure; cultures of the residual, perirenal hematoma yielded positive results for *Escherichia coli* and *Enterococcus faecium* and therapeutic management of the infection included a two‑week administration of cefotaxime and vancomycin.

The other patient presenting with a post‑PCA Clavien–Dindo grade III complication experienced persistent macroscopic hematuria following the PCA procedure, necessitating the placement of a transurethral catheter for 1 day. The patient was discharged from the hospital the following day. However, 5 days later, the patient presented with recurrent macroscopic hematuria accompanied by blood clots requiring cystoscopy for bladder irrigation. A control CT scan revealed the presence of two small pseudoaneurysms, close to the ablated area which were effectively coil‑embolized. Finally, the patient developed fever (38.8°C) and escalating infection markers (CRP 220 mg/L), which were successfully controlled with an antibiotic treatment.

### Radiological follow‑up

**Figure 3:**
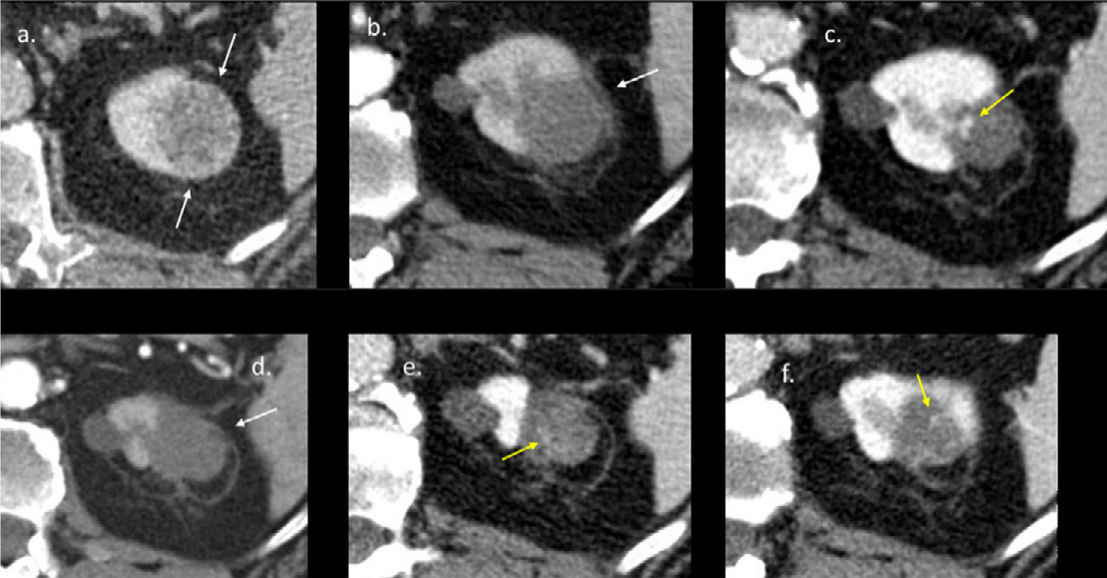
Axial contrast‑enhanced CTs of the patient with two local tumor recurrences. **a.** RCC lesion in the left lower pole before ablation (white arrows). **b.** CT scan 3‑month post‑ablation shows no contrast enhancement in the ablation zone (white arrow). **c.** CT scan 12 months post‑ablation shows contrast enhancement (yellow arrow) in the ablation zone, and thus a local tumor recurrence. **d.** CT 3 months after the second cryoablation shows no residual tumor in the ablation zone (white arrow). **e–f.** On the CT 12 (e) and 18 months (f) post‑ablation the contrast enhancement was very subtle (yellow arrow) and stable. Patient is further followed‑up.

Out of the 68 initially treated renal lesions, 6 of them (9%) showed a persistent viable part of the ablated tumor on the 3‑month follow‑up CT scan; in 1 patient no 3‑month follow‑up CT scan was available and this patient was excluded for early radiological follow‑up analysis. This results in a primary treatment efficacy of 91.0%. Out of the six persistent tumors, three were retreated with cryoablation (Figure 3), two opted for active surveillance, and one switched to immunotherapy. No viable tumor was present on the 3‑month follow‑up CT of the re‑ablated tumors, which increases the secondary treatment efficacy to 95.6%.

**Figure 4:**
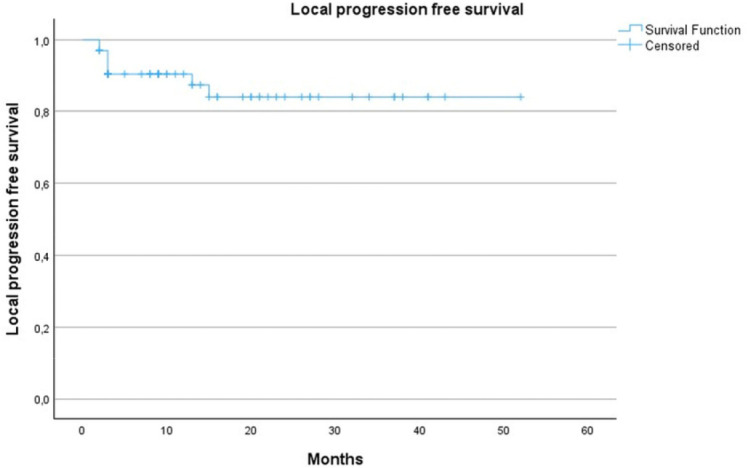
Kaplan–Meier estimate of local progression‑free survival is 87.4% (standard error of 4.8%) at 1 year.

One year follow‑up imaging was available in *n* = 26 patients. Local tumor recurrence occurred in *n* = 2 patients one year following the cryoablation. Estimated LPFS at 15 months was 92.9% (standard error [SE]: 0.48). The Kaplan–Meier curve for LPFS reveals an estimated survival of 87.4% at 1 year. One of the patients with new onset of viable tumor in the ablation area 1 year after PCA, underwent a secondary procedure (Figure 4). Another patient, who underwent secondary PCA due to the persistence of a viable tumor 3 months post‑initial ablation, showed recurrent local tumor progression 6 months following the re‑ablation procedure and was successfully re‑ablated in a third, sequential PCA‑procedure.

### Overall survival

**Figure 5:**
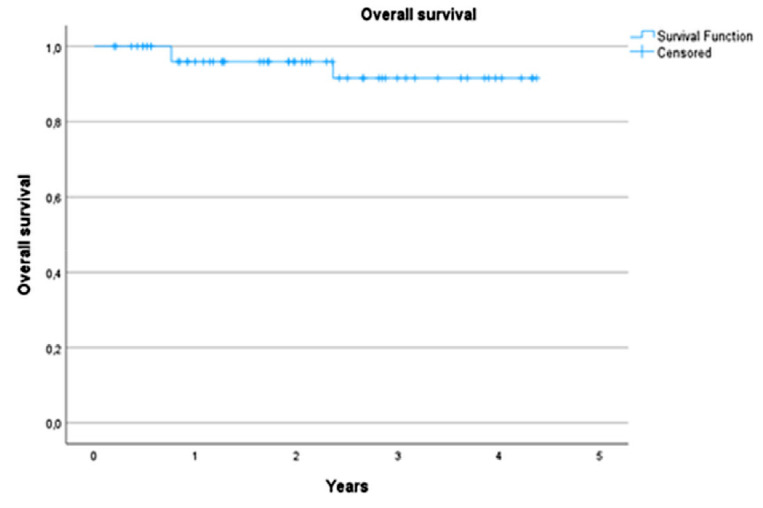
Kaplan–Meier estimate of the OS rates at 1 year was 95.9% (standard error [SE] of 2.8%) and 91.6% (SE: 5.0%) at 3 years.

Three patients (5%) died during the follow‑up period. Kaplan–Meier estimate of the OS rates was 95.9% (SE of 2.8%) and 91.6% (SE: 5.0%) at 1 and 3 years of follow‑up respectively (Figure 5). One patient died related to RCC metastatic disease. This patient had a history of PN in the left kidney and underwent PCA for another RCC mass in the right kidney and one in the partially resected left kidney. Unfortunately, the 3‑month follow‑up CT scan showed one persistent viable tumor in the ablated kidney and also distant tumoral lesions for which palliative immunotherapy was started. Despite systemic immunotherapeutic treatment, the patient developed liver and peritoneal metastases and died 9 months after the index PCA.

## Discussion

Technical success was very high with the complete ablation circle performed per protocol and complete periprocedural coverage of the ice‑ball on CT in 71 out of 72 tumors (98.6%); incomplete ablation was identified in *n* = 1 patient, related to depletion of argon gas at the near end of the PCA procedure. However, no viable tumor was seen on the 3‑month follow‑up in this patient, with no evidence of local tumor progression after 2 years post‑ablation.

The overall complication rate of 10.8% and rate of major complications (Clavien–Dindo grade ≥ III) of 3.1% is in line with other reports: Breen et al. reported a major complication rate of 4.9% over 473 procedures and Garnon et al. found a major complication rate of 3.4% over 647 procedures [8, 17], confirming that percutaneous CT‑guided cryoablation of T1 RCC is safe, with a major complication rate potentially inferior to that of PN as demonstrated in a recent meta‑analysis [5, 22]. In addition, PCA might also be more efficient and safer than other percutaneous, thermal (heat) ablative techniques, including RFA and microwave ablation (MWA) related to the risk of incomplete heat ablation in lesions larger than 3 cm in diameter or in lesions located nearby blood vessels because of the ‘heat sink effect’ and to the risk of procedural surrounding tissue damage respectively [23]. Finally, a significant drop in hemoglobin level after the PCA procedure was found which might be related to the presence of a perirenal hematoma or fluid collection in 20% of the patients. The large majority of these hematomas were small and clinically insignificant with no symptoms and no need for transfusion.

In this study, the primary treatment efficacy was 91.0%, slightly below the range observed in published studies, which is in between 95.3% and 97.5% [9, 11, 16, 17]. This could be related to the presence of a high rate of included patients with a history of previous surgery of partial or radical nephrectomy for the management of RCC. In the study of Hegg et al. including only patients undergoing PCA after PN, a primary treatment efficacy at 3 months imaging of 90.7% was found, which is more in line with our results [24].

Interestingly, in the four re‑ablated tumors, two showed local tumor progression during follow‑up which is in contradiction to the current literature data demonstrating that in all the re‑ablated lesions a complete response on follow‑up imaging was found [9, 24, 25]. Only Spiliopoulos et al. documented a patient who underwent three reinterventions due to recurrence [16]. In addition, the patient with two sequential, local tumor progressions, had no history of previous surgery and no histology was available due to an insufficient sample during biopsy. The other patient with a history of tumor progression after two sequential sessions of PCA over time had a history of radical nephrectomy as treatment of a clear cell RCC.

This study has several limitations. First, this is a single‑center study, including a limited number of patients and treated tumors. Second, related to the retrospective study design, some data are incomplete or missing; however, most of the periprocedural complications and recurrence during follow‑up could be identified based on available angiographic and cross‑sectional imaging. Third, during follow‑up not all patients underwent 3 months, 1‑ and 2‑year CT‑follow‑up related to further follow‑up in the referring community hospitals.

## Conclusion

This study confirmed that CT‑guided PCA of RCC is safe, with a low major complication rate of 3.1%. In addition, a high efficacy rate of >90% of complete tumor destruction after one PCA session, even in a patient sample with a substantial number of patients with a previous history of partial or radical nephrectomy for RCC. Last, this study also shows the potential for PCA re‑intervention after local tumoral recurrence.
